# Supplementary material for: Identification of Transcriptional Regulators of Immune Evasion Across Cancers: An Alternative Immunotherapeutic Strategy for Cholangiocarcinoma
Source: Cancers (Basel). 2024 Dec 17;16(24):4197. doi: 10.3390/cancers16244197 (PMC11674672; doi:10.3390/cancers16244197)
Supplement: Supplementary file 1 [file cancers-16-04197-s001.zip › cancers-3276423-original Western blot images.pdf]

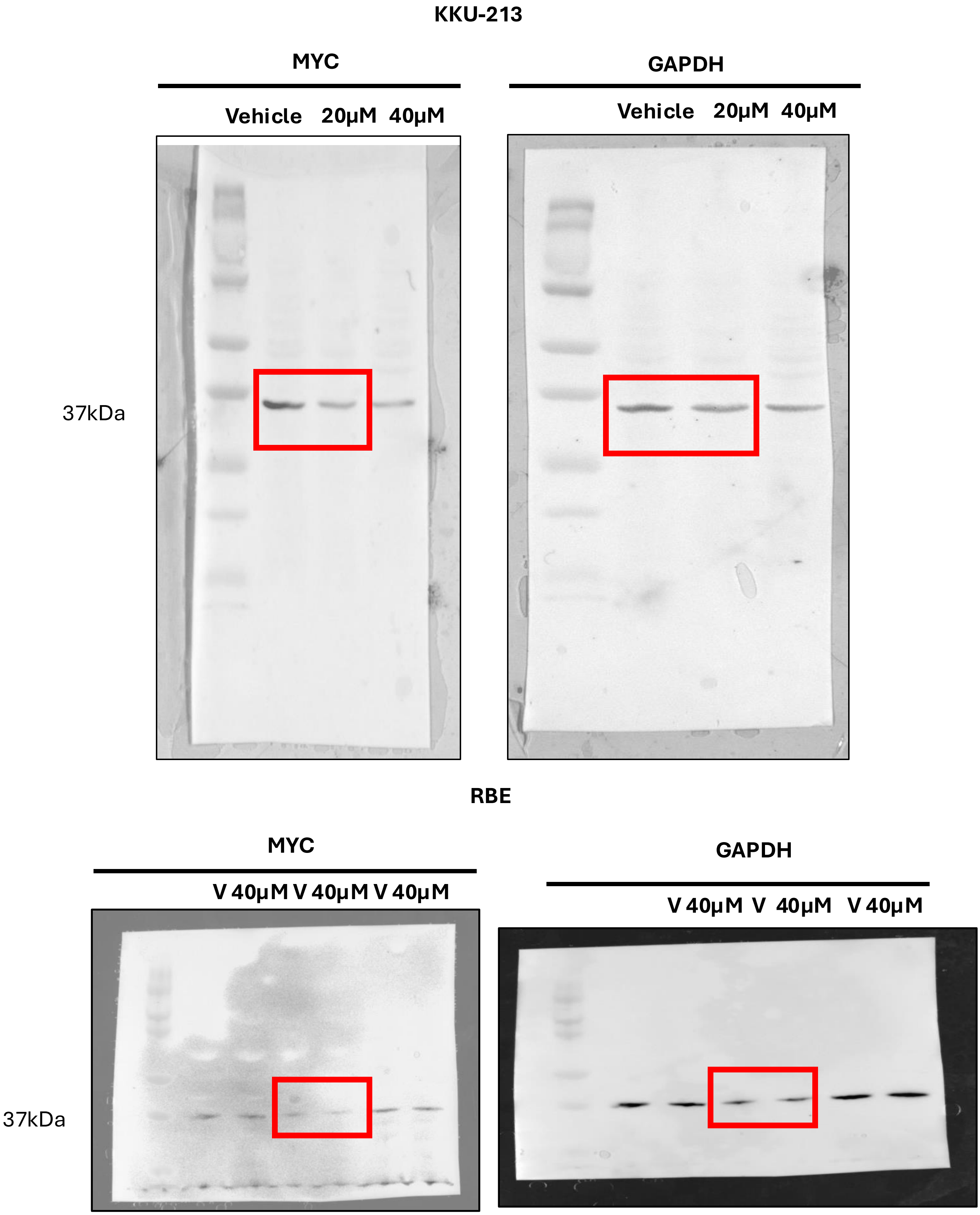

As seen in Figure S9 – Inhibition and Knockdown of MYC in CCA cell lines. A) Western Blot of MYC protein in KKU-213 after MYC inhibitor (10074-G5) treatment. C) Western Blot of MYC protein in RBE after MYC inhibitor (10074-G5) treatment.

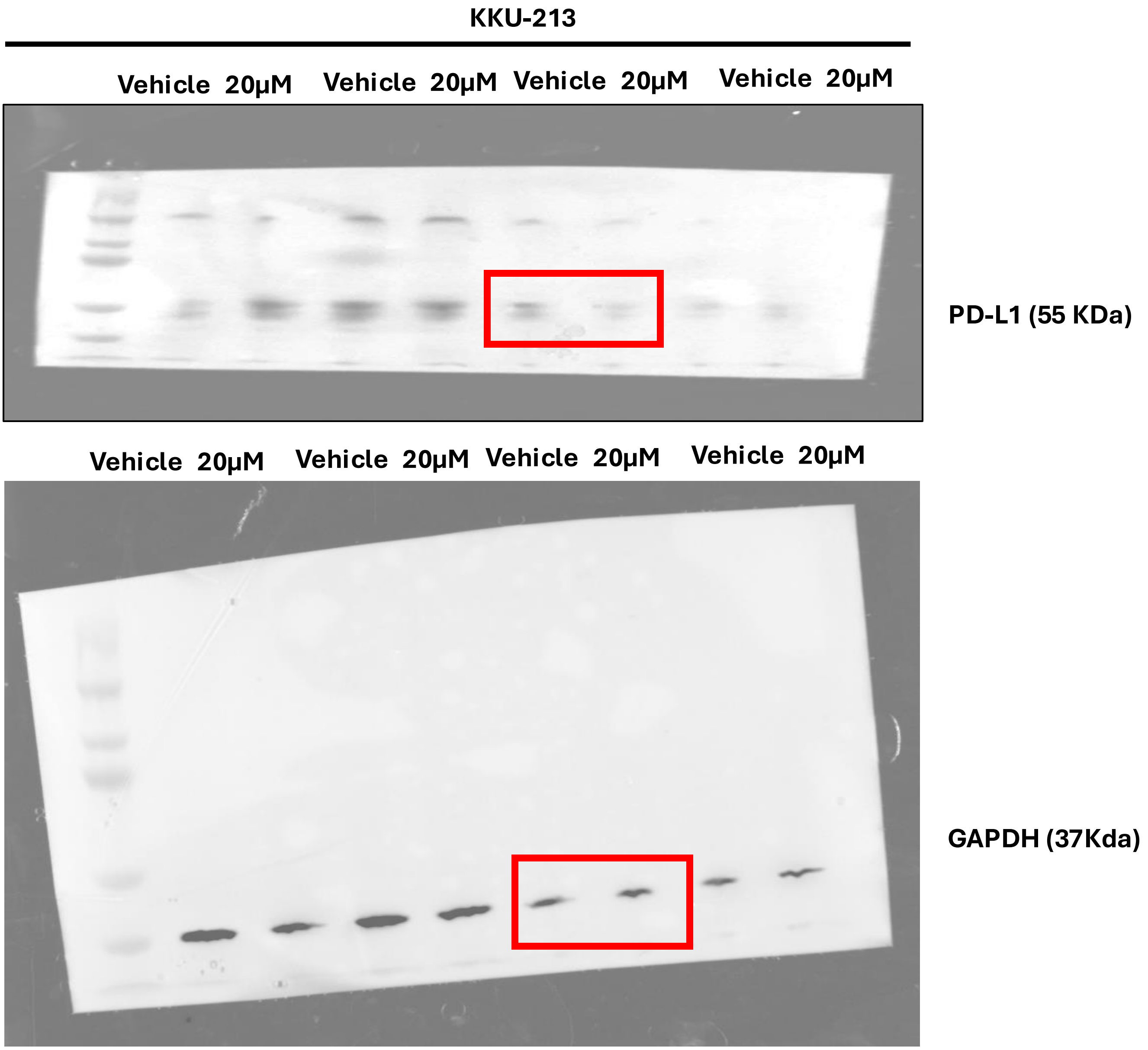

As seen in Figure 4 – Inhibition or knockdown of master regulator results in the downregulation of PD-L1 expression in CCA cell lines KKU-213 but not RBE. a) Western Blot of PD-L1 after MYC inhibitor treatment in KKU-213 cells.

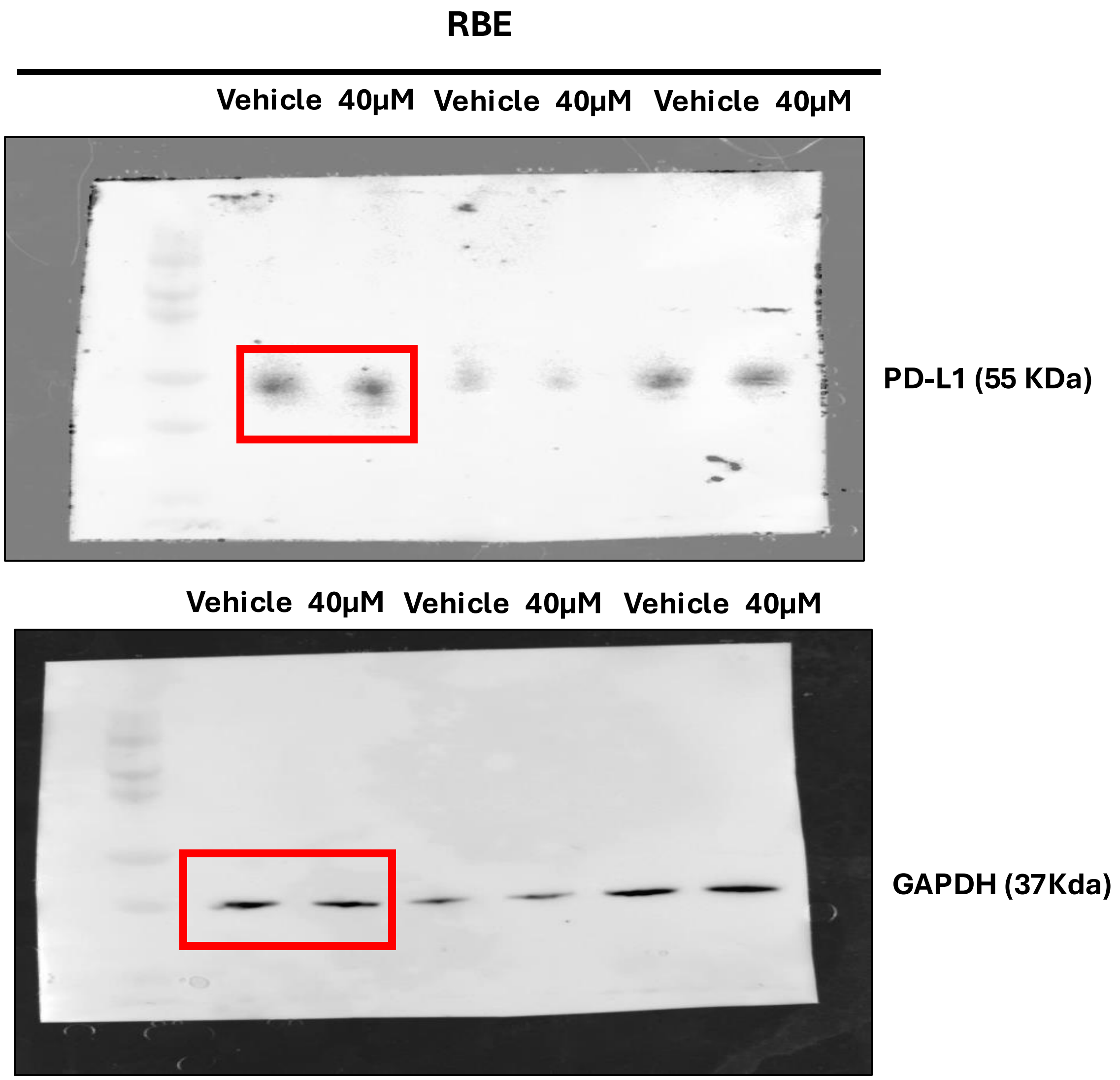

As seen in Figure 4 - Inhibition or knockdown of master regulator results in the downregulation of PD-L1 expression in CCA cell lines KKU-213 but not RBE e) Western Blot of PD-L1 protein after MYC inhibitor treatment in RBE cells
