# Supplementary material for: Identification of Transcriptional Regulators of Immune Evasion Across Cancers: An Alternative Immunotherapeutic Strategy for Cholangiocarcinoma
Source: Cancers (Basel). 2024 Dec 17;16(24):4197. doi: 10.3390/cancers16244197 (PMC11674672; doi:10.3390/cancers16244197)
Supplement: Supplementary file 1 [file cancers-16-04197-s001.zip › cancers-3276423-supplementary figures.pdf]

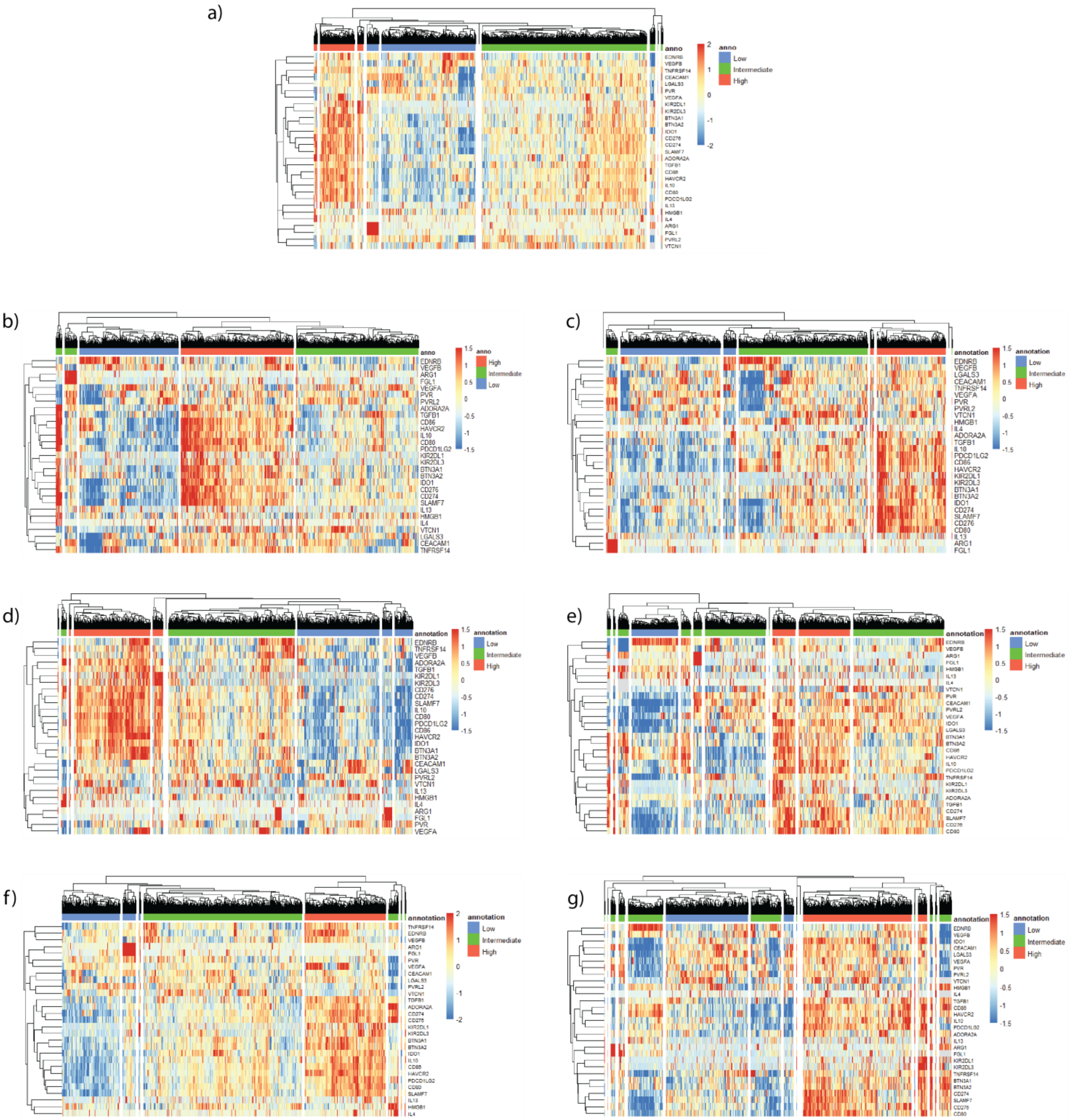

Figure S1. TCGA PanCancer Samples can be stratified according to IC expression. This plot represents hierarchical clustering of PanCancer data sets against immune checkpoint genes. a) Pan cancer b) High Leukocyte Infiltration, c) Low Leukocyte Infiltration, d) High CD4 and CD8 Infiltration, e) Low CD4 and CD8 Infiltration, f) High CD8 Infiltration, g) Low CD8 Infiltration datasets samples.

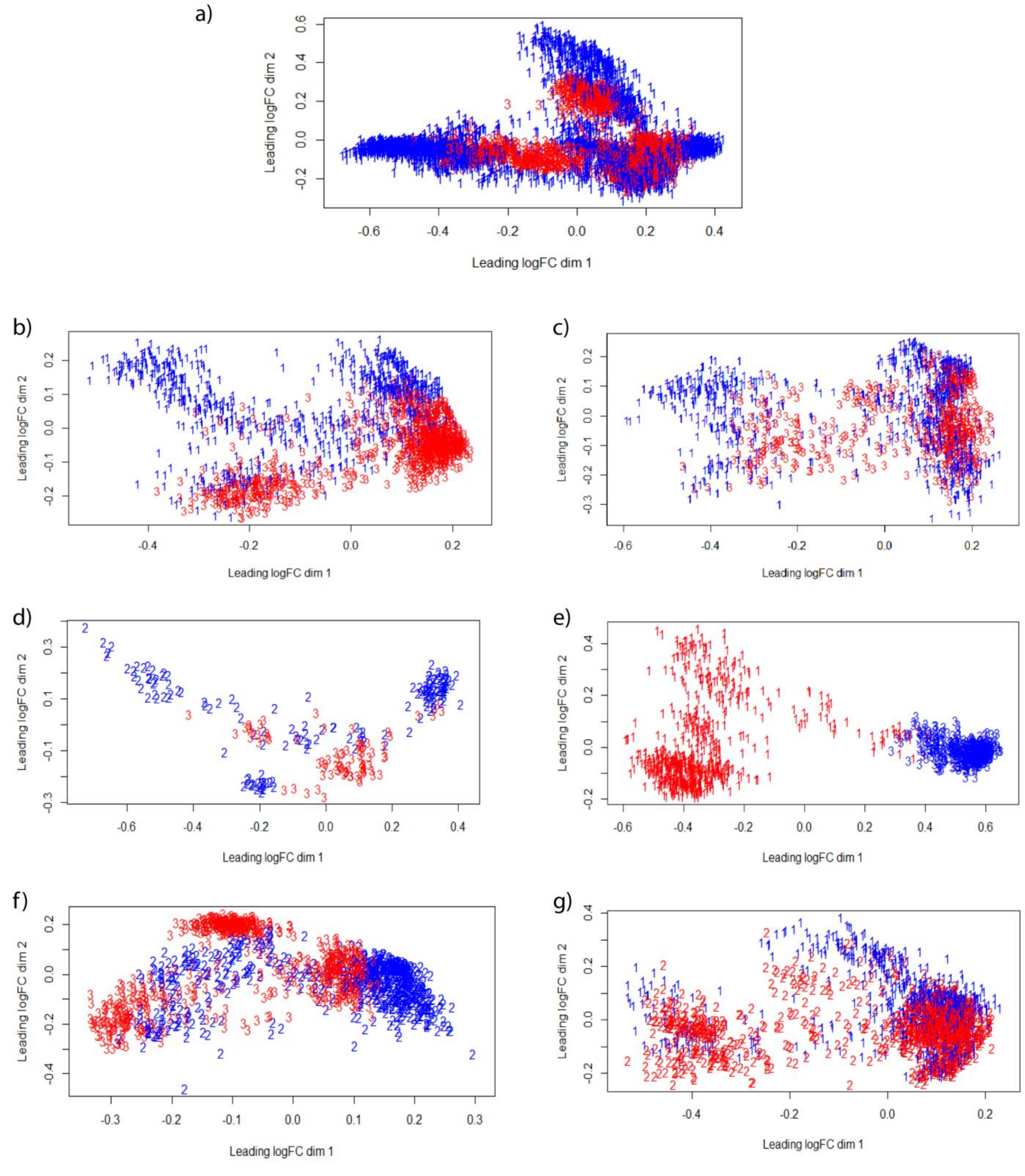

Figure S2. Multidimensional Scaling of a) Pan cancer b) High Leukocyte Infiltration, c) Low Leukocyte Infiltration, d) High CD4 and CD8 Infiltration, e) Low CD4 and CD8 Infiltration, f) High CD8 Infiltration, g) Low CD8 Infiltration datasets samples. The groups low expression (blue) and high expression (red) of co-inhibitory ICs are compared for sample similarity.

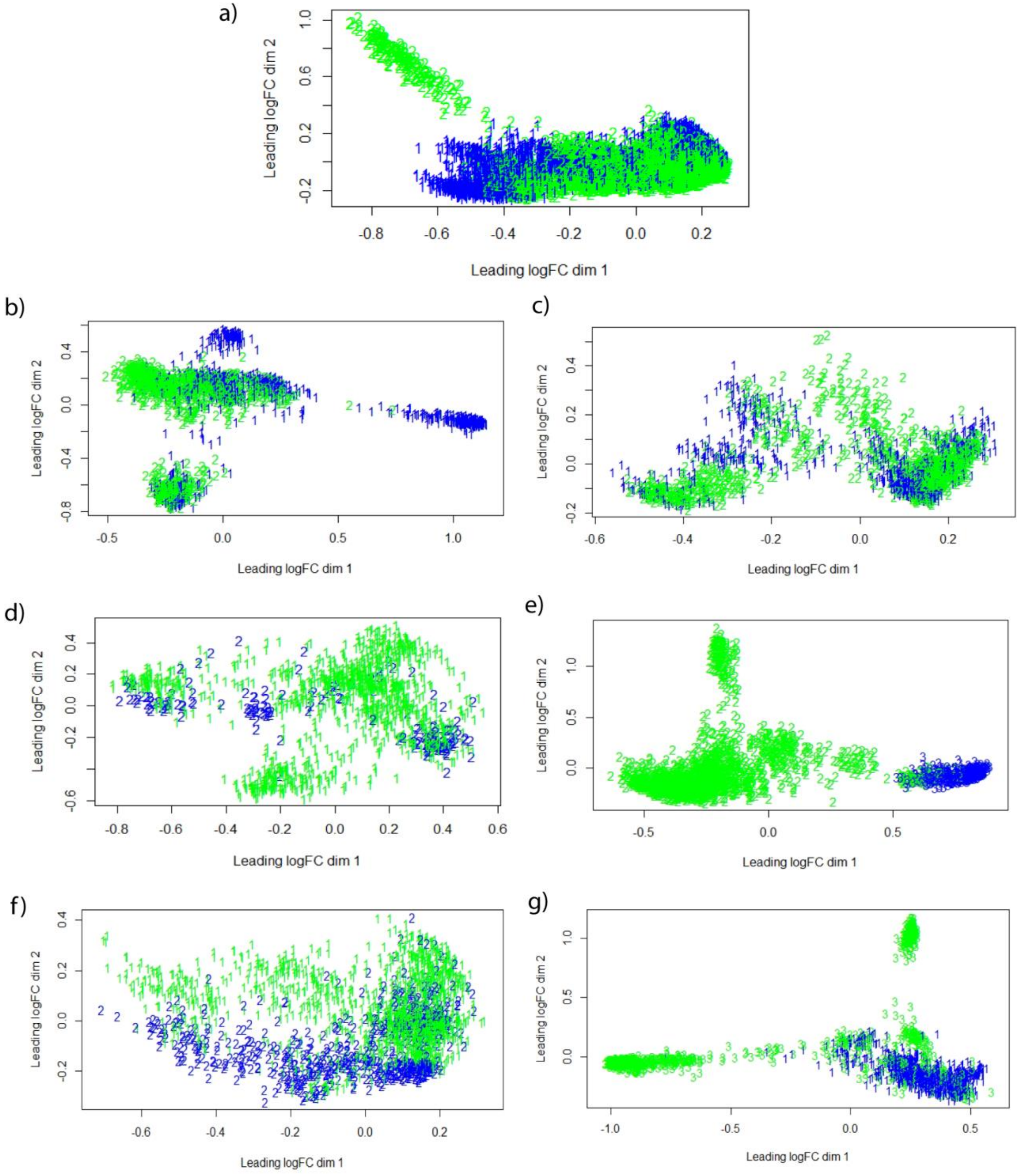

Figure S3. Multidimensional Scaling of a) Pan cancer b) High Leukocyte Infiltration, c) Low Leukocyte Infiltration, d) High CD4 and CD8 Infiltration, e) Low CD4 and CD8 Infiltration, f) High CD8 Infiltration, g) Low CD8 Infiltration datasets samples. The groups low expression (blue) and intermediate expression (green) of co-inhibitory ICs are compared for sample similarity.

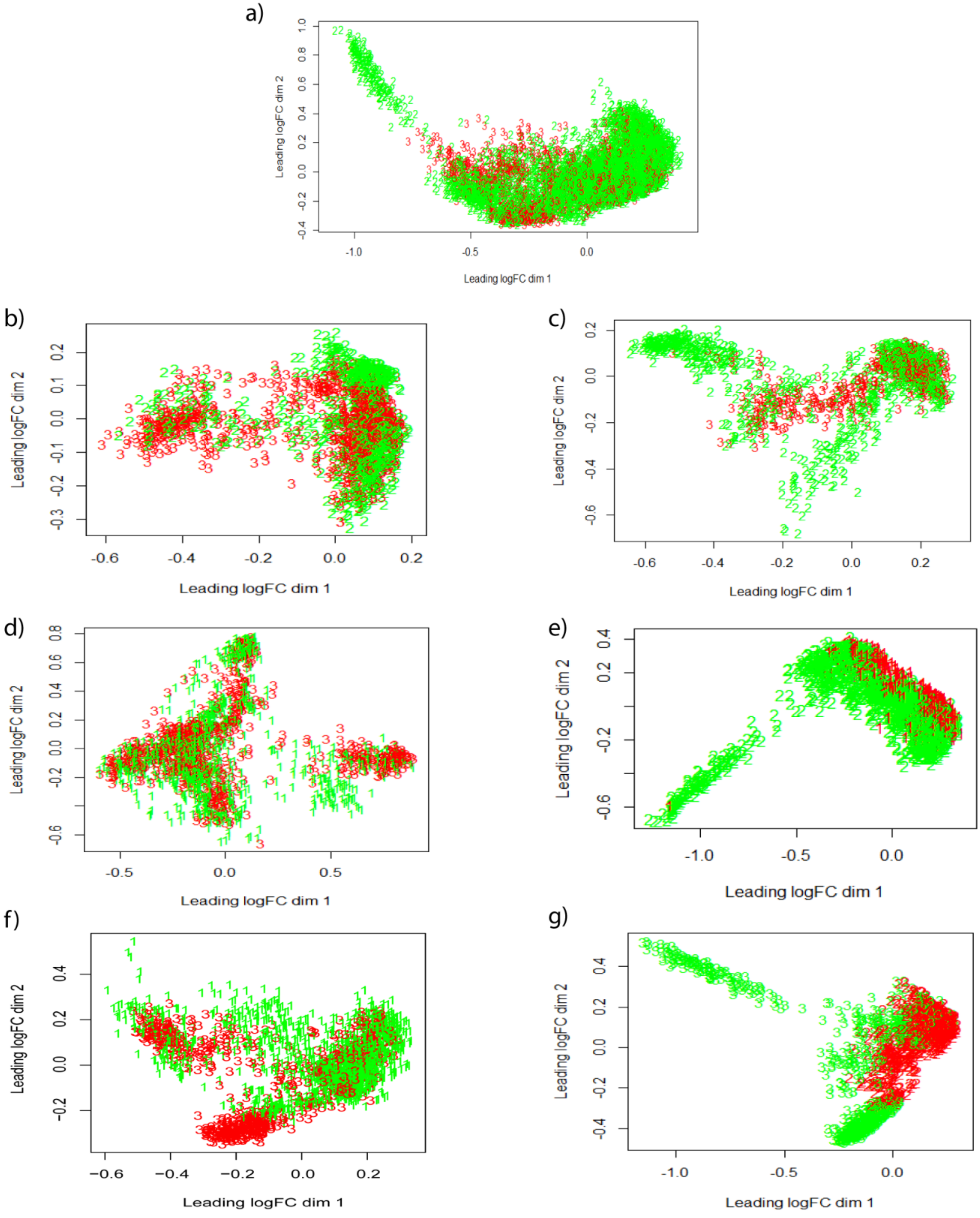

Figure S4. Multidimensional Scaling of a) Pan cancer b) High Leukocyte Infiltration, c) Low Leukocyte Infiltration, d) High CD4 and CD8 Infiltration, e) Low CD4 and CD8 Infiltration, f) High CD8 Infiltration, g) Low CD8 Infiltration datasets samples. The groups intermediate expression (green) and high expression (red) of co-inhibitory ICs are compared for sample similarity.

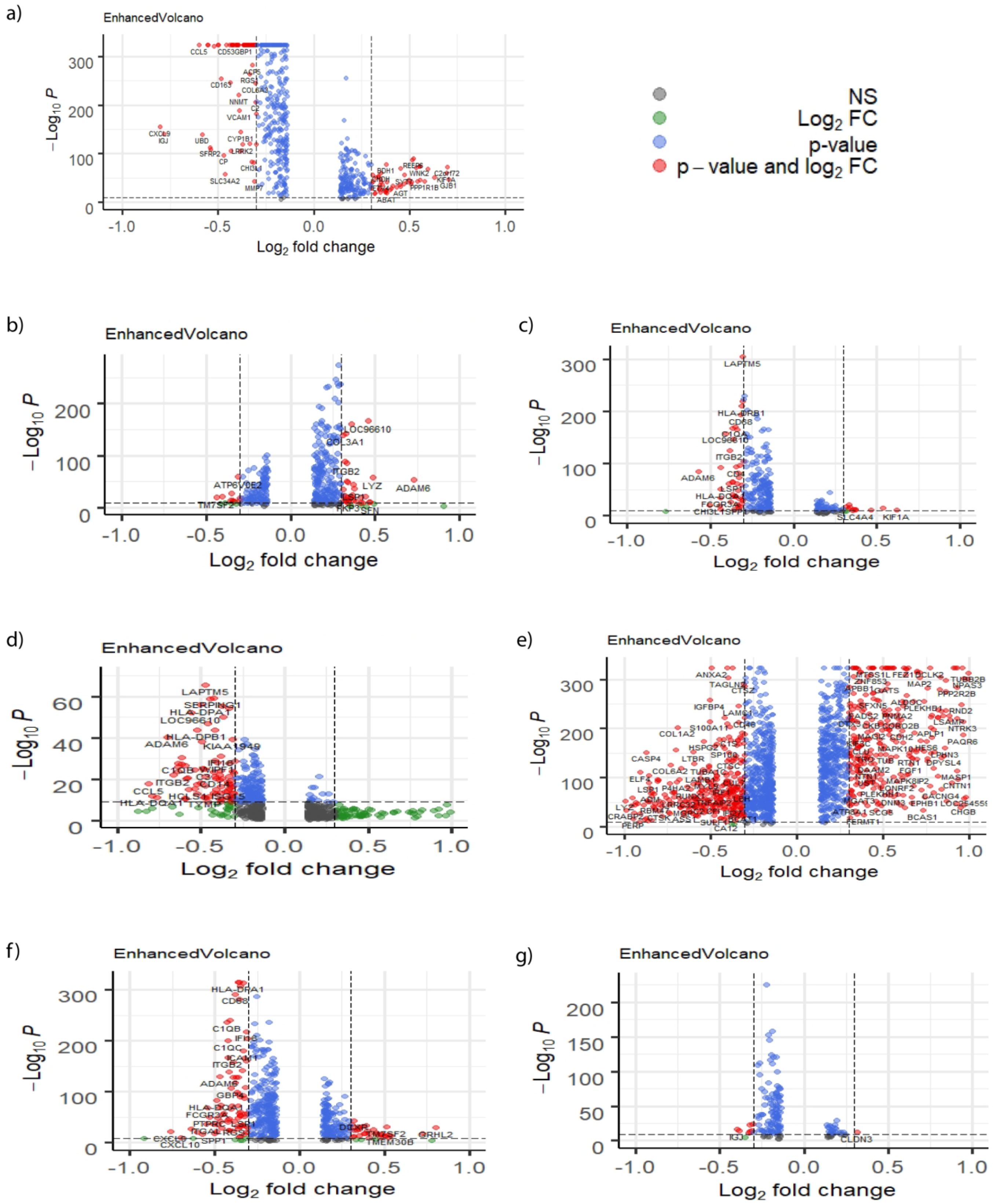

Figure S5. Volcano plots of the comparison between low expression to high expression in the datasets: a) Pan cancer b) High Leukocyte Infiltration, c) Low Leukocyte Infiltration, d) High CD4 and CD8 Infiltration, e) Low CD4 and CD8 Infiltration, f) High CD8 Infiltration, g) Low CD8 Infiltration.

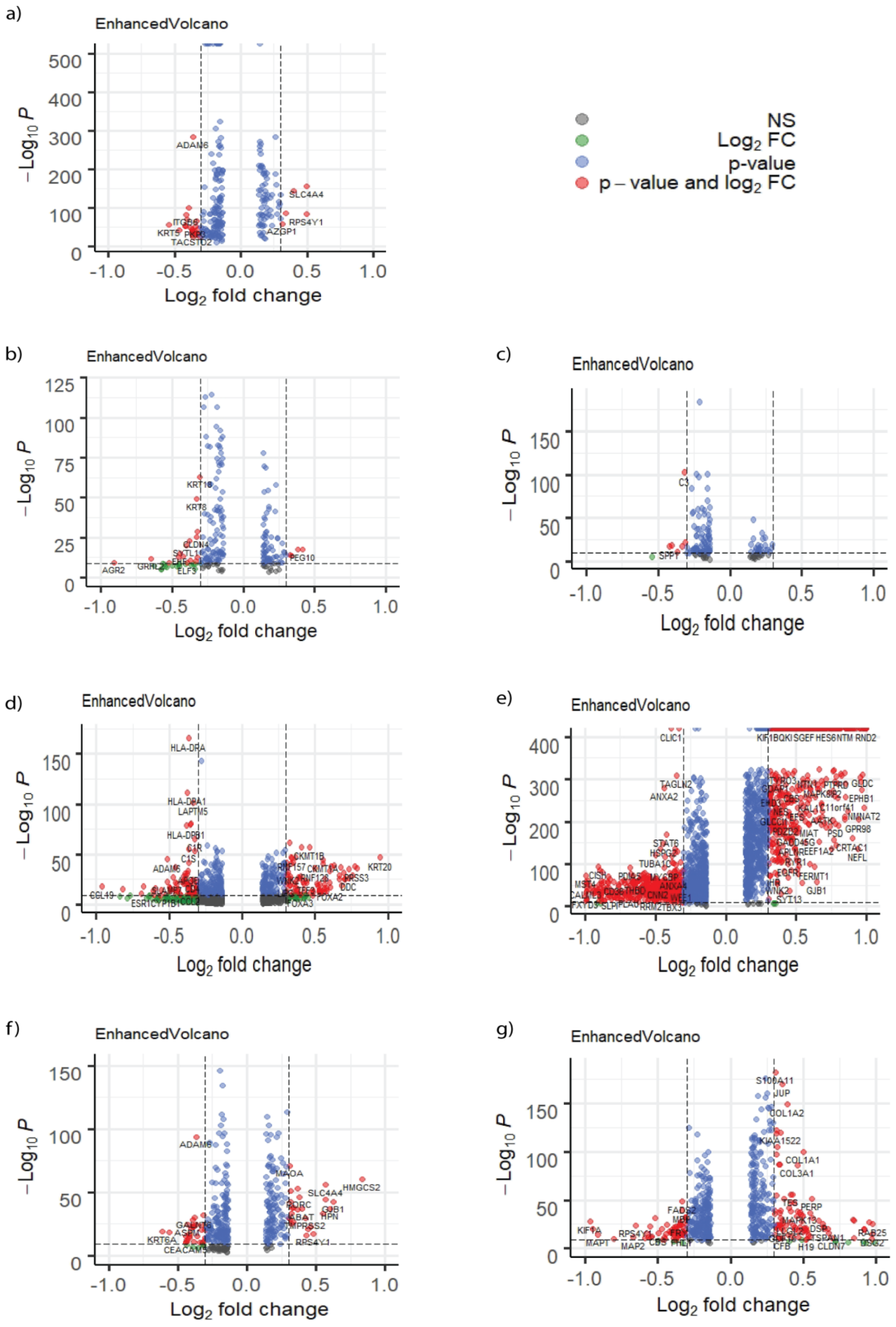

Figure S6. Volcano plots of the comparison between low expression to intermediate expression in the datasets: a) Pan cancer b) High Leukocyte Infiltration, c) Low Leukocyte Infiltration, d) High CD4 and CD8 Infiltration, e) Low CD4 and CD8 Infiltration, f) High CD8 Infiltration, g) Low CD8 Infiltration.

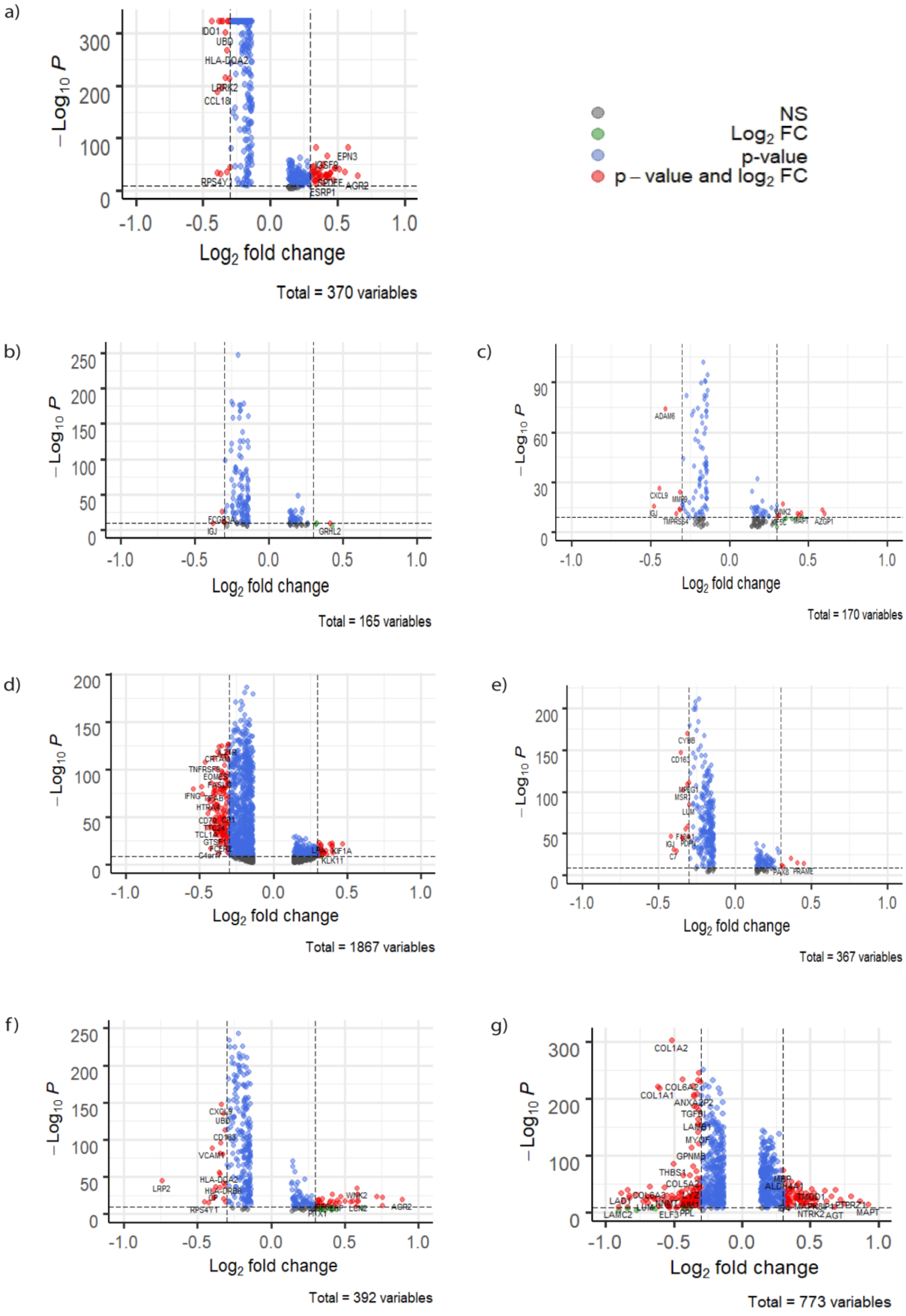

Figure S7. Volcano plots of the comparison between intermediate expression to high expression in the datasets: a) Pan cancer b) High Leukocyte Infiltration, c) Low Leukocyte Infiltration, d) High CD4 and CD8 Infiltration, e) Low CD4 and CD8 Infiltration, f) High CD8 Infiltration, g) Low CD8 Infiltration.

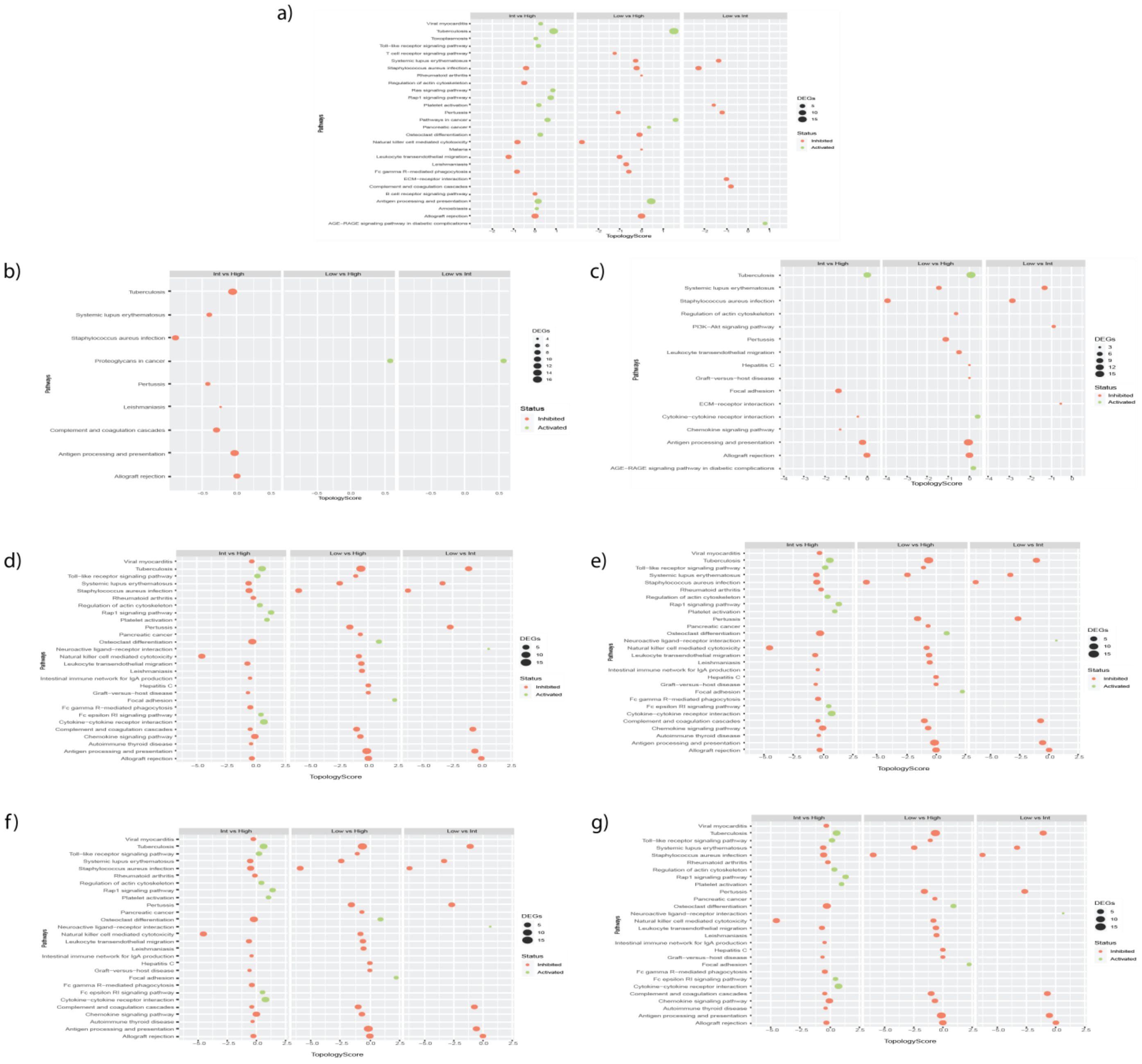

Figure S8. Pathway Enrichment of all subsets and their comparisons. a) Pan Cancer b) High Leukocyte Infiltration, c) Low Leukocyte Infiltration, d) High CD4 and CD8 Infiltration, e) Low CD4 and CD8 Infiltration, f) High CD8 Infiltration, g) Low CD8 Infiltration.

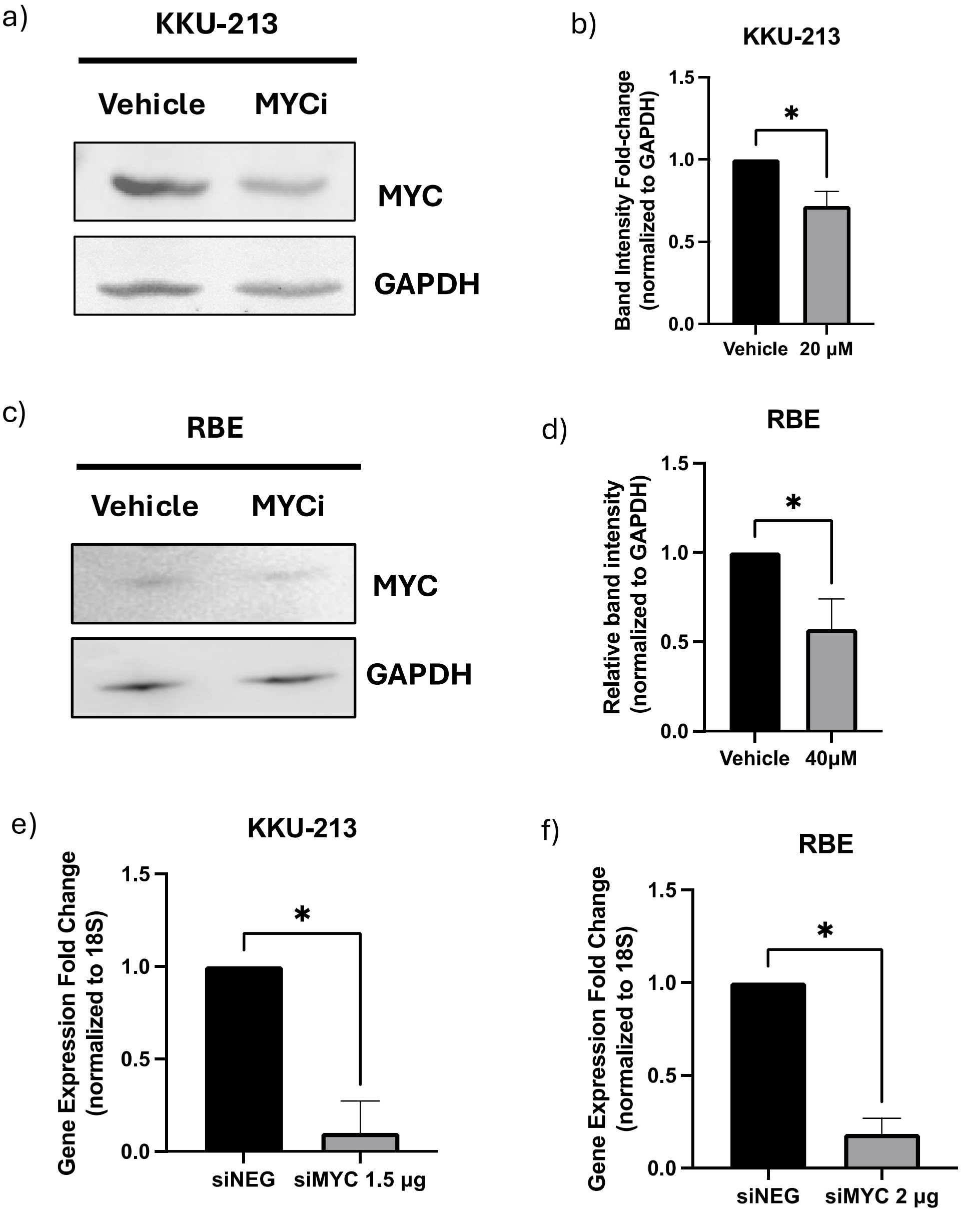

Figure S9. Inhibition and Knockdown of MYC in CCA cell lines. A) Western Blot of MYC protein in KKU-213 after MYC inhibitor (10074-G5) treatment. B) Densitometry analysis of MYC protein normalized against GAPDH band intensity. C) Western Blot of MYC protein in RBE after MYC inhibitor (10074-G5) treatment. D) Denisotimetry of MYC protein normalized against GAPDH band intensity in RBE cell line. E-F) MYC gene expression normalized against 18S RNA after siRNA knockdown of MYC in E) KKU-213 cells and F) RBE cells . Statistically significant differences were deemed at p-value <0.05 (*).

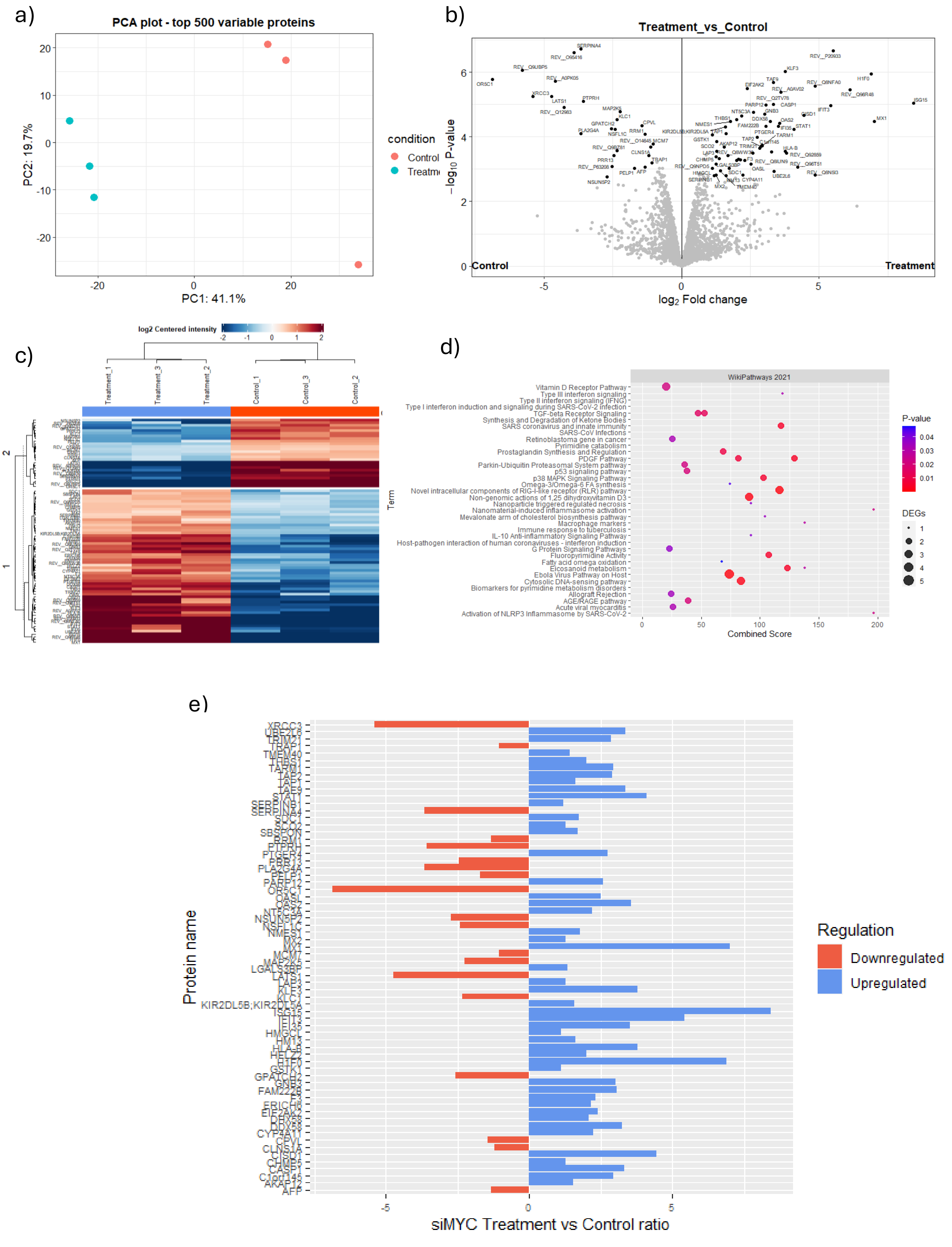

Figure S10. Knockdown of master regulator results in modulation of immune related markers in the cancer cell line proteome in KKU-213 cell line. a) PCA of KKU213 cell samples either treated with siMYC or Vehicle control. b) Volcano Plot of differentially expressed proteins. c) Heatmap of Differentially expressed proteins. d) Pathway Enrichment of differentially expressed proteins. e) Relative expression of Immune related proteins after siMYC treatment compared to control.

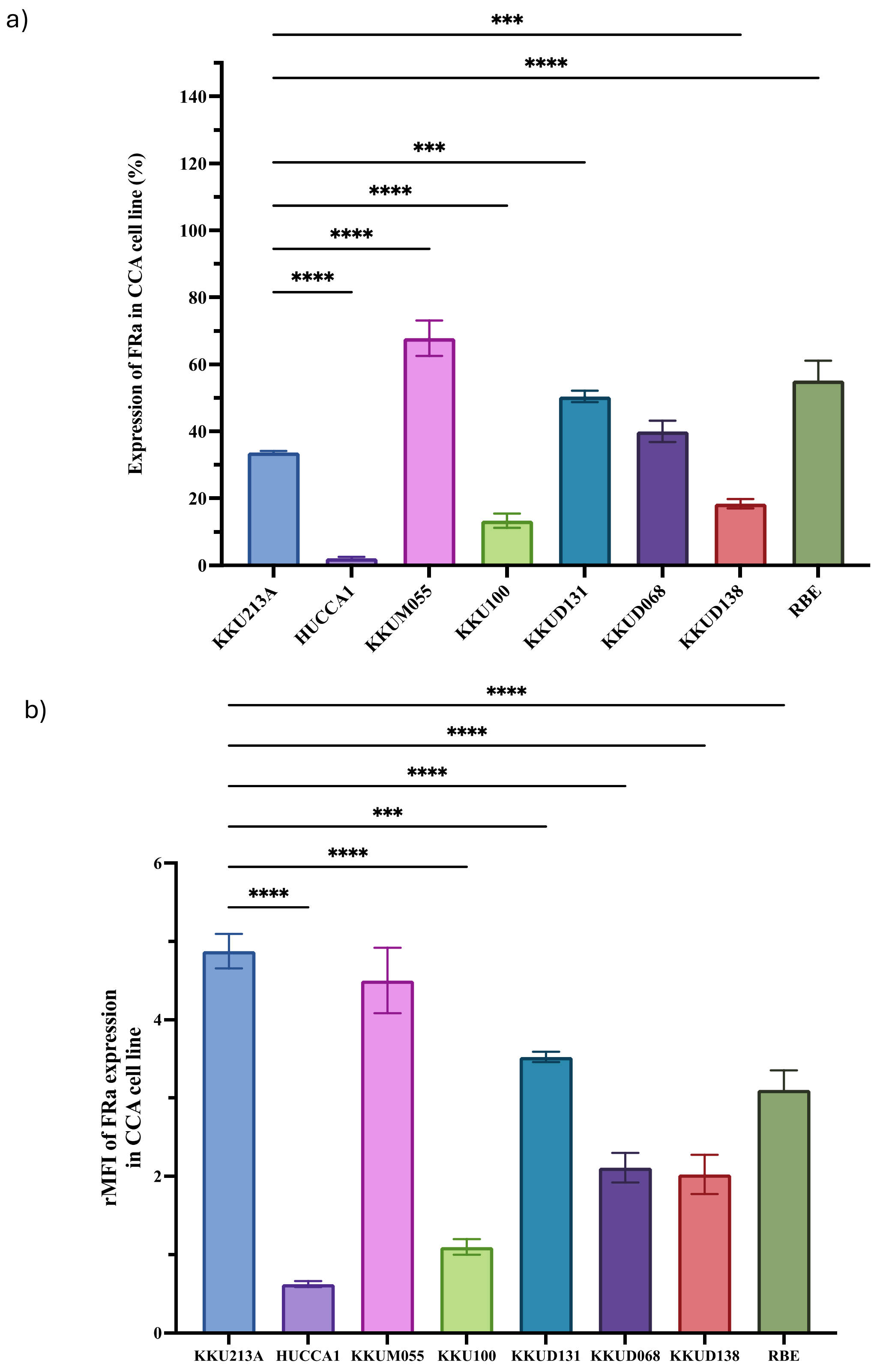

Figure S11. CCA cell lines express FRα. a) Percentage expression of FRα in CCA cell lines. b) rMFI of FRα across CCA cell lines. Statistically significant differences was deemed at p-value < 0.001 (***), 0.0001(****).

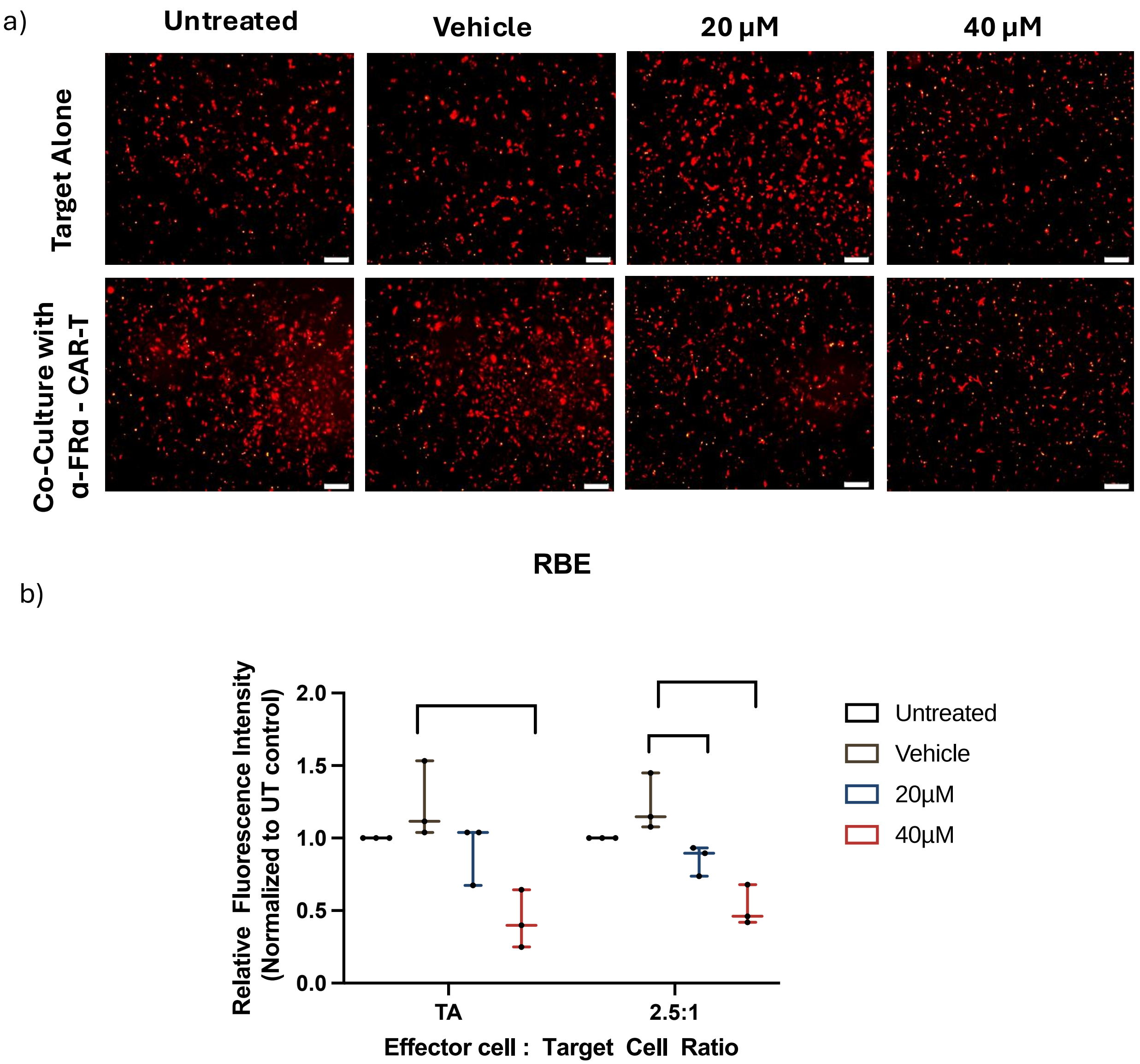

Figure S12. Inhibition or MYC (MR-IE of CCA) does not potentiate immune-mediated cell death through CAR-T cell based therapy in RBE cell line. a) Fluorescent images of RBE tagged with CellTracker Orange treated with MYC inhibitor in varying doses and co-cultured with anti-FR-α CAR-T cells. b) Cell Survival plot compared between target cell alone (TA), and co-culture with anti-FR-α at an effector to target cell ratio of 2.5:1.
